# Understanding and using quantitative genetic variation

**DOI:** 10.1098/rstb.2009.0203

**Published:** 2010-01-12

**Authors:** William G. Hill

**Affiliations:** Institute of Evolutionary Biology, School of Biological Sciences, University of Edinburgh, West Mains Road, Edinburgh EH9 3JT, UK

**Keywords:** genetics, animal breeding, quantitative genetics, heritability

## Abstract

Quantitative genetics, or the genetics of complex traits, is the study of those characters which are not affected by the action of just a few major genes. Its basis is in statistical models and methodology, albeit based on many strong assumptions. While these are formally unrealistic, methods work. Analyses using dense molecular markers are greatly increasing information about the architecture of these traits, but while some genes of large effect are found, even many dozens of genes do not explain all the variation. Hence, new methods of prediction of merit in breeding programmes are again based on essentially numerical methods, but incorporating genomic information. Long-term selection responses are revealed in laboratory selection experiments, and prospects for continued genetic improvement are high. There is extensive genetic variation in natural populations, but better estimates of covariances among multiple traits and their relation to fitness are needed. Methods based on summary statistics and predictions rather than at the individual gene level seem likely to prevail for some time yet.

## 1. Introduction

Traits such as size, obesity or longevity vary greatly among individuals, and have continuously distributed phenotypes that do not show simple Mendelian inheritance.

*Quantitative genetics*, also referred to as the *genetics of complex traits*, is the study of such characters and is based on a model in which many genes influence the trait and in which non-genetic factors may also be important. The framework can also be used for the analysis of traits such as litter size that take a few discrete values, and of binary characters such as survival to adulthood that have a polygenic basis. The quantitative genetics approach has diverse applications: it is fundamental to an understanding of variation and covariation among relatives in natural and managed populations, of the dynamics of evolutionary change, and of methods for animal and plant improvement and alleviation of complex disease.

On the premise that many genes and the environment act and interact to determine the trait, founders recognized that it would be difficult if not impossible to determine the action of individual trait genes. Statistical methods were invented by Fisher (1918) and Wright (1921), the analysis of variance and path coefficients, respectively, to partition the variation and describe the resemblance between relatives, and such tools and methods developed in quantitative genetics have had widespread application in disciplines way outwith their original targets.

The models and summary quantities defined by Fisher and Wright have remained at the heart of the subject not least because they provide ways to make predictions of quantities such as the response to artificial and natural selection. Useful parameters include, for example, breeding value (*A*), which is the expected performance of offspring, and heritability (*h*^2^ = *V*_A_/*V*_P_, the ratio of additive genetic variance or variance of breeding value *V*_A_ to the overall or phenotypic variance *V*_P_, but widely misunderstood). In view of the assumed complexity of the underlying gene action, involving many loci with unknown effects and interactions, much quantitative genetic analysis has, unashamedly, been at a level of the ‘black box’.

Basic questions range widely: what do the genes do; how do they interact; on what traits does natural selection act; why is there so much genetic variation; and can we expect continued genetic improvement in selection programmes? Ultimately, we want to know at the molecular level not just which genes are involved, whether structural or regulatory, but what specific nucleotide change in each gene or alternatively copy number variant is responsible for the quantitative trait effect, and how the genes are controlled. Much progress is being made in addressing these problems, but many questions remain.

For many decades claims have been made that quantitative genetics was dead or dying but, condescendingly, perhaps still useful until the contents of the black box were revealed, a feat which would be ‘just round the corner’. We are indeed becoming increasingly able to peer inside the box and can ask whether our statistical models of genetic variation in traits are so unrealistic that the edifice may topple. Studies have, however, already revealed almost 50 quantitative trait loci (QTL), many identified to genes, segregating for human height (see later); but these QTL, likely to be individually among the most important, contribute only about 5 per cent of the genetic variation. In view of its complexity, it therefore seems likely that the black box will remain cloudy for a while, even though fed information on, inter alia, myriads of genetic markers, levels of gene expressions and trait phenotypes. Statistical methodology which works and is continually developed to incorporate extensive marker and other new data seems likely to remain important for some time yet: better to work with the whole beast rather than try to assemble its parts from inadequate instructions.

I will address some of the background and some of these questions in this personal perspective, which is inevitably uneven in coverage and references, and reflects my interests, biases, knowledge and lacunae. It will focus particularly on animal improvement, an area which has both stimulated many developments in quantitative genetics, and is relevant to the welfare of man. Other recent perspectives and summaries from different viewpoints can be found in, for example, papers by Roff (2007), from the Third International Conference on Quantitative Genetics (2009, *Genetica* **136**, 211–386), and in a *Nature* Insight series (2009, *Nature* **456**, 719–744).

## 2. The statistical foundations of quantitative genetics: models, assumptions and predictions

Let us review the standard assumptions in quantitative genetic analysis, address whether they stand up, and if not how much it matters.

### (a) Partition of variance components

In the model proposed by Fisher (1918) and developed by Cockerham (1954) and by Kempthorne (1954), variances and covariances among relatives are described in terms of the variances in additive genetic effects or breeding values, *V*_A_, interactions of effects between alleles within loci (dominance, *V*_D_) and among loci (epistasis, *V*_AA_, *V*_AD_, …) (Falconer & Mackay 1996; Lynch & Walsh 1998). These partitions are not dependent on numbers of genes or how they interact, but in practice the model is manageable only when the effects are orthogonal, requiring many important assumptions. These include random mating, and hence Hardy-Weinberg equilibrium (i.e. no inbred individuals), linkage equilibrium (which requires many generations to achieve for tightly linked genes) and no selection. Gianola & de los Campos (2008) emphasize these, also providing an elegant formalization for the variance–covariance matrix **V** of phenotypic values of a group of individuals for a single trait:2.1
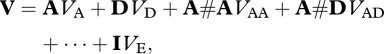
where **A** is the numerator relation matrix, or twice kinship (co-ancestry) of individuals, **D** defines dominance relationships and *V*_E_ the environmental variance. For the epistatic terms, # denotes element-by-element multiplication, but applies only for unlinked loci. Many more terms may be included, such as maternal genetic effects, and genotype × environment interaction. The model has unlimited opportunities for complexity. This is a strength, in that it is all-accommodating, and a weakness, in that datasets may be adequate to allow partitioning into only very few components.

### (b) Linearity

The regression of offspring phenotype on that of parent for the same or different traits is usually assumed to be linear and, equivalently, so is the regression of response on selection differential. This important assumption holds under multivariate normality of phenotypic and genotypic values and thus the central limit theorem assuming multifactorial inheritance. Some traits, such as litter size or lifespan, are clearly not normally distributed, but adequate transformations can be invoked or departures ignored.

### (c) The infinitesimal model

Response to the first generation of selection can be predicted from the breeder's equation *Response* = *h*^2^ × *selection differential*. Selection changes gene frequencies and hence the genetic variance, so predictions of response in subsequent generations formally require knowing individual gene effects and frequencies. Fisher's ‘infinitesimal model’, formalized by Bulmer (1980), provides a practical but biologically unrealistic resolution: infinitely many unlinked genes each of infinitesimally small additive effect, so that selection produces negligible changes in gene frequency and variance at each locus. The within-family or Mendelian segregation variance changes only from inbreeding, and the change in between-family variance (the ‘Bulmer effect’) depends only on the intensity and accuracy of selection practised. Hence the selection response in successive generations can be predicted from estimable base population parameters such as heritability and phenotypic variance, selection practised and inbreeding.

## 3. Developments in statistical methods and applications

### (a) Parameter estimation

Estimates of genetic parameters such as heritability are needed as a basis for description and prediction. Traditional methods such as analysis of variance or regression cannot cope adequately with unbalanced data and the complex pedigrees found outside the laboratory. They have been superseded by more sophisticated methods, often in the context of livestock data (Lynch & Walsh 1998; Sorensen & Gianola 2002), which have been further developed as computing power has increased. An important generalization has been the development of the ‘animal model’ (aka ‘individual animal model’ or ‘individual model’) in which the phenotype of each individual is defined in terms of effects, and the genetic structure is incorporated in the variances and covariances of these effects. For example, a basic model is3.1
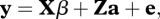
where **X** and **Z** are design matrices, *β* is a vector of fixed effects (e.g. years), **a** is a vector of random effects (breeding values) and **e** is a vector of random errors; and var(**y**) = **ZAZ**’*V*_A_+**I***V*_E_ where **A** is the additive relationship matrix (equation (2.1)). The model is general and flexible: it can incorporate, albeit with increasing computing needs, other covariance terms such as common environment among full sibs, repeat observations, maternal genetic effects (e.g. birth weight dependent also on dam's genotype as a mother) and multiple traits.

In retrospect, a surprisingly recent development has been in the modelling and analysis of longitudinal traits such as body weight which changes over time. The variances and covariances can be described directly by continuous covariance functions (Kirkpatrick & Heckman 1989) or, equivalently, as parameters of random regression coefficients (Schaeffer & Dekkers 1994).

The generality of the animal model and the fact that most field data (whether humans, livestock or natural populations) are highly unbalanced have created a need for sophisticated and general analytical methods. These use restricted maximum likelihood (REML) or Bayesian principles, facilitated by the availability of specialized computer packages (see reviews by those much involved in their development: Thompson 2008; Sorensen & Gianola 2002). Developments continue, stimulated by the need to deal with non-standard data, e.g. on discrete-valued traits, and to incorporate information on multiple marker genes.

The animal model lends itself to analyses of natural populations, where data are on many traits on a limited number of individuals and the relationship structure is complex. Data are obtained from populations that have been studied long term, such as great tits or red deer, and where births and parentage are recorded or deduced to provide pedigrees (see Kruuk (2004) for exposition and papers in *Proc. R. Soc. B* **275**, 593–750, 2008 for examples). Indeed, as genotyping costs fall there are increasing opportunities to expand pedigrees. While relatively simple objectives are to estimate genetic variances and covariances, a broader aim is to use data on breeding success to obtain estimates of the genetic parameters of fitness *per se* (Kruuk *et al*. 2000) and of those characters which determine it, i.e. elements of the selection gradient or partial regression of fitness on each trait. In a natural population, the selection *has* occurred or is currently taking place as a consequence of fitness differences, and a major aim is to infer these selective forces.

The model and methods are flexible but reliable parameter estimation remains a problem and the literature is awash with poor estimates. Few datasets, whether from livestock, laboratory or natural populations, are of sufficient size to obtain useful estimates of many genetic parameters, e.g. there are 30 variances and covariances for four traits when fitting only additive genetic, sib environment and residual effects, let alone say, dominance, epistasis and maternal genetic effects. We all have our pet ideas as to what are important sources of variation or covariation, and fit models accordingly, but typically many different models can fit almost as well (e.g. full sib common environment and dominance). The animal model can cope with selection and assortative mating, but only if the data on which decisions are based is included (e.g. an analysis on a trait of adults if selection is on any trait of juveniles). Animal breeders encounter many such problems, but they are typically more serious for data from natural populations where datasets may be small, poorly structured and include multiple traits. Some traits associated with fitness, i.e. the selection ‘criterion’, may not be recorded, and some individuals may die or leave the population before recording. Hadfield (2008), for example, reviews some of these problems and suggests methods for dealing with them.

### (b) A new approach: use of high density molecular markers in the partition of genetic variance

Very high density of mapping with multi-locus single nucleotide polymorphism (SNP) chips provides a different method to estimate genetic variances. Pairs of full sibs share 50 per cent of alleles on average, but because linked genomic regions are transmitted, the actual proportion shared varies about expectation, with a s.d. of approximately 4 per cent for humans (Visscher *et al*. 2006). Hence, the genetic variance can be estimated *within* families from the regression of phenotypic similarity of sibs for a trait on the actual proportion of genome shared as determined by SNP identity, and is free of confounding by environmental differences between families or maternal genetic effects (Visscher *et al*. 2006, 2007). Estimates of heritability of human height from this method are about 80 per cent consistent with those from traditional methods. The method can be extended to estimate genotype-sharing among members of non-pedigreed natural populations (including fish), if there is enough money to buy the chips, but relatives providing the most information such as sibs may also share environments.

### (c) Prediction of breeding value (or genetic merit) from phenotypic data

Prediction of breeding values is a fundamental component of modern breeding programmes, as those with the highest values should be selected. The major unifying development, Best Linear Unbiased Prediction (BLUP), is due to Henderson (1950, 1984) and incorporates both fixed (environmental) effects and random (genetic) effects in a mixed model (see e.g. Lynch & Walsh 1998; Sorensen & Gianola 2002). As computing power has increased, the animal model (equation (3.1)) is now used, enabling simultaneous prediction of breeding values for all traits of individuals differing in age, location, numbers of records and numbers of relatives. As all selection candidates can be compared at frequent intervals, with overlapping generations it is possible to cull and select continuously.

BLUP is best in the sense of minimum variance among linear predictors, but only if population parameters are well estimated. It is unbiased in that, as more data are accumulated, the predicted breeding values approach the true values; and while it allows for selection, requires the important but often unachievable proviso that *all* information on all traits on which selection is practised is included in the data. Further, if any selection is practised, the infinitesimal model assumption is implicit (but often forgotten) in the use of the relationship matrix **A** to quantify variances and covariances across generations.

## 4. The statistics in practice: investigating and informing the assumptions

Many major assumptions are made in the applications of quantitative genetics, but the issue is not the formal correctness of models used, rather the extent to which they work reasonably well. There is not space for a full review, but more discussion and examples are given elsewhere (e.g. Falconer & Mackay 1996; Lynch & Walsh 1998; Walsh & Lynch 2009). We first consider quantitative data at the whole trait level before considering information from studies of QTL and genes.

A major problem is to obtain data of adequate structure and quantity. For example, in the infinitesimal model all genetic variation is assumed to be additive. In random mating populations it is, however, usually impossible to estimate epistatic variances with any precision because the coefficients are very small and highly correlated with those of non-epistatic components (e.g. **A** and **A#A** matrices in equation (2.1)). These in turn may be confounded with other parameters, such as genetic maternal effects to explain why, say, a daughter-dam correlation exceeds twice that of half sibs in the absence of epistasis. Linkage disequilibrium (LD) is patently present, but that owing to close linkage is assumed absent in the infinitesimal model. The orthogonality assumptions in equation (2.1) may not hold, but how should that be tested? Hence, much of the evidence based on quantitative information is unsatisfactory in being so inconclusive, for example in failing to reject even the infinitesimal model as the following examples show.

In a classical study Clayton *et al*. (1957) found good agreement between heritability estimates from different sources and with predictions of selection response. Sheridan (1988), however, showed that there are frequently wide differences between selection responses predicted from base population parameters and those actually realized, but his analysis failed to take into sufficient account the sampling errors of the predictions or the responses (Walsh & Lynch 2009, ch. 14). It is a common observation that regressions of progeny on parent phenotype are roughly linear, but in detailed studies failures can be found (e.g. Gimelfarb & Willis 1994). Frankham (1990) has shown that selection responses for fitness-associated traits are generally asymmetric, faster down than up, as might be anticipated with a previous selection plateau. We have tried direct application of the infinitesimal model predictions using REML/BLUP to mouse selection experiments, but with inconsistent results: for example a rather poor fit for feed intake in one line (Meyer & Hill 1991), but an excellent fit despite a four-fold change in body fatness in another (Martinez *et al*. 2000). Under the infinitesimal model, the pattern of response in finite populations is predictable from base population parameters. Using data summarized by Weber (2004) on responses at generation 50 relative to those in the first generation, we showed that ‘realistic’ models based on distributions of gene effects, including some of the large effects, provided a good fit to the data; but an infinitesimal model (including mutation) fitted almost as well (Zhang & Hill 2005*a*). Perhaps, this robustness is unsurprising: Barton & de Vladar (2009) show that the population dynamics can be modelled well using approaches from statistical mechanics, where the population is described solely in terms of stationary distributions of gene frequencies and continued response is insensitive to the details of the genetic architecture.

**Figure 1.**
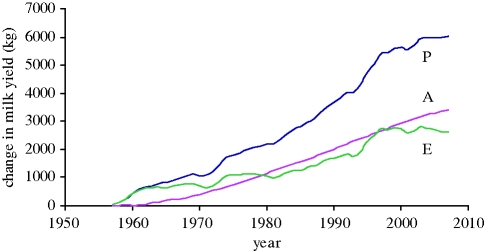
Changes in milk yields of US Holstein cows: phenotypic mean yields (*P*), mean breeding values (*A*) and environmental effects (*E* = *A* − *P*) derived from USDA data. Results are given relative to 1957, when the mean yield was 5859 kg. (Adapted from http://aipl.arsusda.gov/eval/summary/trend.cfm).

I am not aware of any ‘experiment’ in which a combination of say REML and subsequent BLUP predictions has been formally tested *in vivo*. Hence, let us take a pragmatic view: if something works in practice is that not sufficient even if the theoretical foundations are generally unsubstantiated? For over 30 years BLUP and related methodology have dominated genetic evaluation of dairy cattle, and models have become increasingly complex. The spectacular genetic improvement achieved is illustrated in figure 1 and is in accord with the infinitesimal model and BLUP prediction.

So while the genetic models adopted may be very crude, their generally satisfactory behaviour explains why many scientists and practitioners applying quantitative genetic principles do not lose much sleep over model assumptions. We are, however, getting new kinds of information from studies at the individual QTL or gene level which should inform, improve, or in due course may replace the classical models and methods. The path from primary gene effect to phenotype may be complex, however; increasingly so as more genes are involved. Even when the genetic lesion is known, using that information to effect a ‘cure’ may be far from straightforward, as the work with cystic fibrosis shows (Pearson 2009).

## 5. Numbers of genes, their effects, their actions and interactions

Since the time molecular markers became available, extensive studies have been undertaken on analyses to identify QTL and, on occasion, the actual gene or nucleotide (QTN). Indeed, this has been the big quantitative genetics industry of the last two decades. The basic methods are to use associations generated by linkage or LD between marker genes and the trait to locate QTL or to identify and locate mutations having a phenotypic effect and a molecular signal, such as transposable elements. Linkage studies (Lander & Botstein 1989; Haley & Knott 1992) have been conducted in designed studies using crosses of inbred lines or, for example, breeds, and family studies in humans. In view of the few recombinants generated in any region of the genome, the linkage studies are usually unable to provide precise location of QTL in the genome even when many markers are available, and in many cases have not been conducted on a sufficient scale. The availability now of dense SNP maps enables and requires data for analysis in which many generations of recombination between markers and QTL may have occurred to enable fine-scale mapping. In the laboratory, recombinant-inbred lines (RIL) have been developed from crosses of multiple inbred lines to introduce much initial diversity (Chesler *et al*. 2008) and multi-line segregating populations established from inbred crosses have been generated (Valdar *et al*. 2006). As for inbred line crosses, the RIL have the further benefit that animals of identical genotype can be generated and many traits studied in relevant specialized laboratories to make the best use of development time and costs. Association mapping using LD enables high-precision mapping in humans, livestock and natural populations, but requires large datasets and high-density SNP marker panels to be effective. Further, it enables inferences to be drawn about frequencies and effects of genes actually segregating in populations. In view of the large resources needed, it is not surprising that most of the information so far generated from association mapping is on human disease; but these and other traits recorded in such studies, for example height, are already providing an important source of information for all quantitative geneticists.

There is an extensive literature on the basic methodology of QTL mapping (e.g. Lynch & Walsh 1998; Weller 2009) and, for example, Mackay *et al*. (2009) summarize both methodology and achievements. There are many statistical problems involved, even in the most basic QTL mapping studies. Not least is the problem of trade off between power of detection and type-I error, with very extreme significance thresholds having to be set when searching over all the many possible sites in the genome. Hence, the QTL most likely to be found are those of largest effect; very many are likely to be missed; and the estimated effects of those detected are likely to be biased upwards and their position poorly located.

### (a) Some examples

Rather than attempt to review or even summarize the field, I shall just give some examples of the results from the use of different techniques, roughly in descending order of precision, that both provide information and generate questions.

In a summary of the analysis of around 600 P-element insert lines in *Drosophila melanogaster*, a method permitting precise location, Mackay (2009) found that about 17 per cent of the insertions affected sensitivity to the inebriating effects of alcohol (even *Drosophila* have an excuse) and 34 per cent affected locomotor behaviour to a stimulus; and she noted that similar screens have found 22 per cent of insertions affecting abdominal and 23 per cent affecting sternopleural bristle number. Some have large effects, however. In view of the fact that such a high proportion of sites are targets, it is not surprising that there is extensive pleiotropy. Mackay also notes that many show epistatic effects. Similarly, for a range of behavioural traits in mice, in a study of over 200 gene knockout lines, 19 per cent showed abnormal open-field activity (Flint & Mott 2008).

Heterogeneous stocks established by crossing inbred mouse lines can allow fine-scale mapping. In an analysis of 97 traits, including body weight and many biochemical variants, of 843 QTL detected and mapped to within 3 Mb, only 10 individually contributed more than 10 per cent of the variance for any trait and none over 3.5 per cent for body weight or length (Valdar *et al*. 2006). A plot of the distribution of QTL contributions to variance shows a peak at about 2 per cent, though it is likely this is, in effect, a truncated exponential-shaped distribution, as smaller ones are non-significant and missed. In principle, such distributions (obtained also in other studies) can be extended to smaller effects, but some prior distribution must be assumed.

The association studies undertaken with combined samples of 10 000 or more humans are revealing a substantial number of QTL that have been cross-validated and in many cases identified to specific genes. Visscher (2008) and Weedon & Frayling (2008) provide summaries. Some 44 independent variants that affect stature, none of which are rare in the population, have been mapped; but none individually explain over 0.5 per cent of the phenotypic variance. The heritability of the trait is about 80 per cent, and overall only about 5 per cent of the variance has so far been accounted for. None of the variants show evidence of departure from additive gene action, i.e. dominance or epistasis, and the difference between homozygotes is about 0.8 cm (or a little over 0.1 phenotypic s.d.). Although the causal genes have not yet been proven, there is a strong candidate in over half the cases. Of these, many are components of signalling pathways known to be important in skeletal growth and development, demonstrated for example by gene knock-outs in mice (Weedon & Frayling 2008).

For cattle, in July 2009 there were 1375 QTL curated into the database (the cattleQTLdb, http://www.animalgenome.org/), and likely others were discovered by companies but not entered. These were from 83 publications and represented 109 different traits (but many have pleiotropic effect), representing a major effort and expenditure. The number of animals involved in each analysis are far smaller than in the association studies in humans, although data are used from segregation within individual sires who have progeny-tested sons with accurate estimates of breeding value. As only few of the QTL have been finely mapped, there is uncertainty about which of those mapped in different studies to similar genomic regions are the same or different genetic lesions, and how many are false-positives. In a few cases in livestock the actual genes, all having large effect, have been identified and sequenced. Some were already known as major genes, such as double muscling in cattle, for which the myostatin gene has been identified as causative, and others were initially discovered in mapping studies, for example DGAT, which influences milk composition of dairy cattle (see for example Hu *et al*. 2009 for more examples and references). It is not clear yet if there is any general pattern about what genes will be found to act, but clearly some of the large effects are segregating.

## 6. Conclusions on architecture and the ‘missing’ heritability

The different kinds of analysis are revealing that many loci contribute to quantitative genetic variation. This finding is no surprise to quantitative geneticists because the polygenic and specifically infinitesimal models of quantitative genetics have been shown to work so well in prediction, in distributions and in describing long-term selection response, and the more optimistic expectations in early days of QTL mapping of finding a few regions contributing most of the variation was unrealistic. Indeed predictions made by, for example, Robertson (1967) of contributions of increasingly many genes of increasing small effect have generally been borne out.

While the most reasonable hypothesis to explain why most of the genetic variation in human height is not accounted for by the 50 or so loci contributing most is that there are many more, perhaps thousands, of small effect and more extreme frequency, concern has been expressed about the ‘missing heritability’ and various hypotheses proposed (Maher 2008). One is that previous estimates of the heritability are biased by environmental correlations, another that various interactions are responsible. But both are refuted by the within-family analysis of Visscher *et al*. (2007, see above) which gives similar estimates of heritability, shows no evidence of interactions across chromosomes, and a distribution of variance contributed roughly proportional to chromosome length. Rare variants including rare copy-number variants could explain some of the variation, as these would contribute to the estimates of within-family variance, but their effects would be hard to detect with the current resolution of SNP chips. Transient epigenetic effects could contribute to heritability estimates from close relatives (Slatkin 2009), but cannot be a predominant feature as they would not contribute to long-term selection responses.

Perhaps human height is exceptional, for it has a very high heritability and near additivity of variance. Recent association studies on other traits are, however, also revealing many regions of the genome associated with disease risk: almost 20 for type II diabetes (Donnelly 2008), and for schizophrenia, also highly heritable, as significance thresholds attached to individual markers detected in one subset of data were reduced, increasingly more risk could be accounted for in independent sets of cases (Purcell *et al*. 2009). Therefore, the current sample sizes available for genome-wide associated studies are not sufficiently powered to detect the majority of the associated variants.

Neutral genes have an expected U-shaped frequency distribution, *f* (*p*)∝[*p*(1 − *p*)]^−1^, under rare mutation drift balance (Wright 1931), such that if they are additive the variance is contributed uniformly across gene frequencies. Mutant genes under natural selection, either because they have pleiotropic effects on fitness or are subject to stabilizing selection, show a distribution more heavily weighted to extreme frequencies (Wright 1931; Zhang & Hill 2005*a*), such that the variance contributed may also be U-shaped. Such loci are hard to detect in association studies even if they have large effect, partly because they contribute little variance and partly because SNP markers that have intermediate frequencies cannot have high correlation in frequency (*r*^2^) with a rare QTL. The hypothesis that most of the missing variation is associated with extreme frequencies is not, however, supported by the schizophrenia study (Purcell *et al*. 2009).

Another important property to be revealed from such studies is the magnitude of pleiotropic effects of genes on other traits. In view of the large number of height genes already revealed but counting for 5 per cent or less of the variance overall, there must be so many genes affecting it overall that pleiotropy for other traits must be widespread. This accords with the findings of Mackay (2009, see above) from mutagenesis studies. In contrast, in an extensive linkage-based line analysis of mouse skeletal measurements, Wagner *et al*. (2008) concluded that pleiotropic effects were rare. But they set significance thresholds at the same high values for detecting pleiotropic effects as for initial detection, such that even a QTL with exactly the same large effect on each trait would be significant for only a few.

**Figure 2.**
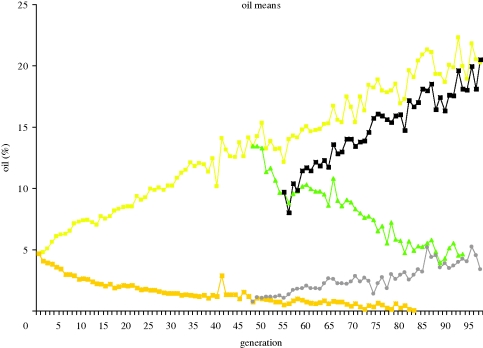
Responses to selection for oil content in maize in the Illinois selection lines. Line designations: IHO (light yellow square), continued selection for high oil, ILO (dark yellow square), for low oil; RHO (green triangle), RLO (white circle), reverse selection; SHO (black square), re-reversed selection. (Adapted from Dudley & Lambert 2004).

A quite different source of evidence on the role of multiple genes comes from the analysis by Laurie *et al*. (2004) of the Illinois maize experiment (see figure 2, discussed further later) with selection for high and low oil content in the kernel. From a line cross made at generation 70 and maintained by random mating for 10 generations to reduce LD, they estimated that about 50 QTL contributed to the response, none exceeding about 0.3 per cent oil to the line divergence of 17 per cent oil. Furthermore, the QTL acted essentially additively with each other and similarly in pure lines and crosses.

Some of the data provide puzzles about how genes act on quantitative traits which no doubt will take some unravelling. In contrast to the extensive QTL association-based mapping studies in humans showing additive gene action and the useful properties of the infinitesimal model, some studies in livestock, plants and laboratory animals have revealed dominance and epistatic interactions (e.g. Carlborg & Haley 2004; Mackay 2009). Can these data be squared?

Interactions, which are second-order effects, are likely to be tiny and very hard to detect if the main effects are already small. Further, unless all the interacting genes are at intermediate frequencies, they are expected to contribute mostly additive variance simply on statistical grounds (Crow 2008; Hill *et al*. 2008). In inbred line cross or mutagenesis experiments, those loci of large effect that can generate most interactions are more likely to be observed than in outbred natural populations, where their heterozygosity is low if they have any deleterious effect on fitness. So we should not necessarily infer wholly additive effects from additive variance.

In view of geneticists' success in unravelling the control of developmental pattern, it would seem straightforward to figure out how the overall size of the organism is controlled. But we now know that many more than 50 genes affect stature, and arguably all 20 000 genes affect all quantitative traits, together with other controlling factors in the genome. So how in the body is the phenotype determined? One can see a role for systems biology, but I am pessimistic about the rate at which the systems will be disentangled: understanding models for connecting tens of interacting genes may be feasible, but not for 1000. So while we will get a lot more information, I do not believe the essentially statistical approach, enhanced by the use of genomic information to mark genomic regions, is on its last legs.

## 7. Maintenance of variation in quantitative traits

Let us turn now from considerations of how quantitative traits are determined to trying to explain why they are so variable in natural and derived populations.

The magnitude of variances and heritability is a property of that population and environment, as it depends on the frequency and effects of the segregating genes, but for the same trait or type of trait they tend to be roughly similar, not just across populations but even across species. Heritabilities (*h*^2^) tend to be highest for conformation traits and mature size, typically 50 per cent or more, and lowest for fitness-associated traits such as fertility (e.g. Mousseau & Roff 1987; Falconer & Mackay 1996; Lynch & Walsh 1998). Conversely, the ‘evolvability’ or genetic coefficient of variation (CV_A_ = *h* × CV), is typically higher for fitness-associated than conformation traits (Houle 1992). Estimates of variance for fitness itself are hard to obtain, but the laboratory-based estimate of *V*_G_ for log fitness is 17 per cent (Fowler *et al*. 1997) and although life-history traits in natural populations show clear evidence of genetic variance, their heritabilities are low (e.g. Kruuk *et al*. 2000). We still seek adequate explanation of what determines levels of quantitative genetic variation, why there is some consistency across populations and species, and why there is so much for fitness-associated traits despite the inference from Fisher's fundamental theorem of its loss by natural selection.

### (a) Mutation and genetic variation

Estimates of the amount of genetic variance contributed by mutation, generally expressed as the ‘mutational heritability’, *V*_m_/*V*_E_, show a surprisingly narrow range over many traits and species, centred about 0.1 per cent (Keightley & Halligan 2009), and equivalent to an increment in CV_A_ of *ca* 0.3 per cent for a trait with a CV of 10 per cent. If all genes were neutral with respect to fitness, *N*_e_ = 250 would maintain a heritability of one-third at the equilibrium *V*_A_ = 2*N*_e_*V*_m_, but unsurprisingly this close relationship between heritability and population size is not found as many mutations are deleterious.

The most studied model for natural selection acting on the trait directly is stabilizing selection, i.e. intermediates fittest. Under this model, genes of large effect contribute more variance when segregating, but have lower expected heterozygosity, and so the predicted variance maintained is proportional to the total mutation rate to trait genes and inverse of the strength of selection (i.e. curvature of fitness surface). If few loci are assumed to affect the trait and typical estimates of the strength of selection are assumed, the predicted variance is much lower than that observed (Turelli 1984). But even if hundreds of genes affect any single trait, the model is not rescued because mutants are likely to have pleiotropic effects on many traits and overall be under stronger selection than on the target trait alone. The finding of segregating genes at intermediate frequencies affecting human height, for example, indicates that selection pressures are weak, and so both population size and selection set upper bounds to the variance maintained (Bürger 2000). Disentangling selection on multiple traits is difficult or impossible; indeed, there is little evidence for stabilizing selection and as much for its converse, disruptive selection, in the summary of published results by Kingsolver *et al*. (2001).

An alternative model is to assume that the selection does not act on the target trait directly but is through pleiotropic effects of the mutant (Keightley & Hill 1990). This does not, however, resolve the dependency of *V*_G_ on population size, nor explain the constancy of trait means. Various aspects of the fit to the data are enhanced both by assuming that the mutants are (nearly) additive for the trait but (partially) recessive for fitness, and worsened by assuming that there are substantial pleiotropic effects on other traits and overall fitness (Zhang & Hill 2005*b*).

There are a plethora of other models, invoking spatial and/or temporal variation in the environment, competition for resources and (even) heterozygote superiority, but none are clear winners. Johnson & Barton (2005, p. 1419) put it well: ‘We are in the somewhat embarrassing position of observing some remarkably robust patterns, that are consistent across traits and species, and yet seeing no compelling explanation for them.’ It is not yet clear how the new genomic data will help, in view of the many genes identified for height, for example. Indeed, the theory requires some rethink to account for the large number of small effects and pleiotropy, and put to best use the new genomic, proteomic and other data that become available.

Further, how the level of phenotypic or environmental variance and hence *h*^2^ are determined has been less studied and is even less well-understood than that of *V*_G_. Evolution of *V*_E_ requires genetic variation of phenotype given genotype, for which there is strong evidence in *Drosophila* (Mackay & Lyman 2005) and in livestock populations (e.g. Sorensen & Waagepetersen 2003). Under stabilizing selection genotypes expressing less variable phenotypes are fitter, leading to evolution to reduce *V*_E_. We have suggested two models that would lead to a balance: an ‘engineering’ cost in resources to obtain and maintain homogeneity; and/or most mutations disrupt the phenotype and tend to increase *V*_E_ (Zhang & Hill 2008), for which there is some evidence (Baer 2008).

## 8. Looking to continued selection response and genetic improvement

Let us consider how genomic and individual QTL or gene information can be used in improvement programmes, and what are the opportunities for continued response using straightforward selection on the quantitative trait and incorporating other technology?

### (a) Using individual quantitative trait loci

There has been extensive theoretical analysis and simulation to develop methods for using individual QTL in plant and livestock breeding programmes by marker-assisted introgression of a QTL from another population or by marker-assisted selection to increase frequency of a segregating gene in the population (e.g. Weller 2009). Clearly, its effectiveness depends on the real effect of the QTL, the relation between the predicted and the real effect, the closeness of available markers to the QTL (obviously best if the actual gene is known), and on its frequency in the population; and its impact will be the greatest when phenotypes are absent (e.g. sex-limited traits) or of low heritability.

Much effort has been expended on QTL detection and on theoretical analysis of how best to incorporate them in improvement programmes. We have much less information on actual effectiveness because much is within commercial companies and conventional selection on continuous traits has continued alongside. In two recent reviews on applications in plant breeding, Collard & Mackill (2008) and Hospital (2008) suggest that the great opportunities have not yet been fully realized. In a comprehensive review on work in livestock, Dekkers (2004) concluded guardedly that ‘The current attitude to marker assisted selection is one of cautious optimism’. I consider that the returns from the extensive R&D on QTL identification in livestock have been low, both because selection responses have been high from conventional selection (e.g. figures 1 and 2) and because estimates of QTL effects and genome location are poor for the lowly heritable traits that are hardest to improve by selection.

### (b) Using genomic selection

The availability of marker panels of thousands of SNPs does, in contrast, appear to be bringing in a real paradigm shift following the pioneering study of Meuwissen *et al*. (2001), and seems likely to be less of a false dawn than the use of individual QTL (or indeed of transgenics). The objective is to predict the breeding values of candidates for selection not by identifying just a few QTL of large effect but, by densely marking the whole genome, to incorporate most variants using historical LD in the population. This information is used to assess sharing of genomes of relatives and to weigh the marker genotypes according to the phenotypic effects associated with each region and the imprecision of estimation of these effects. In view of the close linkage, the LD between markers and genes is unlikely to change rapidly over generations, such that it may be possible to use much less dense marker panels after the initial evaluation (Habier *et al*. 2009).

Development of methodology continues, particularly of the statistical methods required to undertake the BLUP predictions. One approach is to replace the expected relationship matrix **A** (equation (2.1)) by the realized relationship matrix as assessed using high-density markers (Hayes *et al*. 2009). Another is to more overtly make use of possible differences among genomic regions in contribution of variation in the trait, but if it is assumed that the variance in the trait associated with each SNP is sampled from the same normal distribution, the methods are equivalent (Goddard 2009; Hayes *et al*. in press) and can be used by extension of BLUP methodology, ‘genome-wide BLUP (GWBLUP)’. Under the assumption that a limited proportion of the genome contributes most of the variation, selective procedures have been developed, initially by Meuwissen *et al*. (2001), to identify these regions using a Bayesian analysis with some assumed prior distribution of the of number and effects of QTL; but choice of the prior remains controversial.

The methods have widespread potential applications in breeding programmes and can incorporate any number of traits and availability of phenotypic records. Benefits are most obvious in the improvement of sex-limited traits, such as milk or egg production, where young sires have to be selected on the basis of their ancestors' and female sibs' records, and all full brothers have the same predicted breeding value. With the genomic information, the Mendelian sampling contribution to each individual son can be predicted. While more research is clearly needed to optimize methodology, genomic selection is now being introduced in widespread commercial practice, a rapid uptake of ideas first published less than 8 years ago (Meuwissen *et al*. 2001).

The USDA provided the first set of genomic breeding values predicted by GWBLUP for bulls in the USA in January 2009. By making BV predictions for bulls using only data available on their sires, comparisons between predictions with and without the use of genomic information could be made using these bulls' actual progeny performance. For milk yield, for example, the predicted and observed accuracies using just ancestral phenotypic data were 0.35 and 0.32, and by incorporating the genotypic data, the respective figures increased to 0.69 and 0.56 or 0.58 according to whether differential weights were given to different genomic regions (van Raden *et al*. 2009). In the context of dairy cattle improvement, such near doubling in the accuracy of selection is spectacular. Other studies have shown increases in accuracy, but not all as high as expected, for example on a pedigreed population of mice (Legarra *et al*. 2008). Although these need to be understood, for example in terms of numbers of SNPs, the prospects are high, but we await outcomes.

The ideas of genomic selection can be applied to predict disease risk in humans or among selection candidates in livestock, using information on genome sharing with close or more distant relatives (Wray *et al*. 2007). The basic assumption is that many loci contribute to risk, as borne out by analysis at least for schizophrenia (Purcell *et al*. 2009). Perhaps this way, personal genotyping will yield benefits if analysis is put in the hands of those understanding the statistical methodology and its limitations.

Genomics is not the only ‘omics that may provide important information on quantitative traits, and there are alternative ways to use genomic data, such as non-parametric methods (Gianola & de los Campos 2008) that do not use all the Mendelian information. Major developments in the technologies and their use will surely be made. For example, gene expression arrays yield data on thousands more ‘traits’, each individually susceptible to quantitative genetic analysis, and some may well be relevant to particular objective traits. Again some caution is required: physiological predictors of performance, e.g. use of hormone levels, have been much mooted but produced little of practical benefit in livestock improvement. So, overall, it is a question of ‘watch this space’: the extensive new data should be of value for incorporation as ‘markers’ and also new understanding of the biology will be important in its own right and should lead to more effective breeding programmes.

### (c) Maintaining selection response, genetic improvement and evolutionary opportunities

We see the striking changes that have been produced in quantitative traits by selection, for example among breeds of dogs in body weight and behaviour, and in the productivity of modern livestock and crops. Can we expect continued change?

The Illinois maize selection for high and low content of oil in the kernel has been continued since 1896. The low lines have reached a plateau (almost 0% in the low oil line, and presumably at the minimum for seed viability in the low protein line), but the upward lines have continued responding for 100 generations (i.e. years, figure 2). Large and continuing responses have been seen in other laboratory experiments spanning 100 or more generations (Hill & Bünger 2004). Genetic change in crop plants can be estimated by comparing varieties released in different years grown contemporaneously from stored seed. Trials show that there was a steady increase of approximately 1 per cent in the yield of maize in the USA per year of introduction over a 70-year period since 1930 (Duvick *et al*. 2004).

Results for a limited number of generations are shown for cattle in figure 1, but the most intensive continuous selection in livestock has been practised in broiler chickens since the 1950s when specialist meat and egg lines were developed. Responses from selection based primarily on individual phenotype have been enormous (table 1), showing an approximately five-fold increase in 56-day body weight between 1957 and 2001 (Havenstein *et al*. 1994, 2003). Comparisons using modern and old diet formulations showed that at least 80 per cent of these differences were genetic. Responses were continuing at similar rates during the decade since 1991, other than in fatness, where selection to reduce fat had been effective (table 1). Intensive selection on specific traits has led to unfavourable changes in other characters, typically those that are associated with fitness, such as fertility in dairy cattle and leg strength and viability in poultry. Selection pressure has increasingly been put on such traits, such that in broilers viability and leg quality has improved in recent years (McKay *et al*. 2000; Havenstein *et al*. 2003; Hill & Zhang 2009). What these show is that the breeder has to be cognizant of all important traits; but if appropriate selection pressure can then be exerted, a change in direction can be effective, as the Illinois maize lines illustrate (figure 2).

**Table 1.** Comparison of weight at eight weeks and body composition in two trials, the first of 1957 control and 1991 commercial and the second of 1957 control and 2001 commercial broilers reared on a diet using typical specifications of that year. (The difference *D*_1_ denotes changes between 1957 and 1991 and *D*_2_ between 1957 and 2001, and *D*_2_ − *D*_1_ is the estimated change between 1991 and 2001. (Adapted from Havenstein *et al*. 1994, 2003; G. A. Havenstein 2008, personal communication))

year of population	1991 trial			2001 trial			difference
	1991	1957	*D*_1_	2001	1957	*D*_2_	*D*_2_ − *D*_1_
body weight (kg)	3.11	0.79	2.32	3.95	0.81	3.14	0.82
carcass weight (kg)	2.07	0.50	1.51	2.81	0.48	2.33	0.82
carcass yield (%)	69.7	61.2	8.5	74.4	60.8	13.6	5.1
breast yield (%)	15.7	11.8	3.9	21.3	11.4	9.9	6.0
carcass fat (%)	15.3	9.4	5.9	15.9	10.6	5.3	−0.6

It is not surprising that such continued responses are found, as in many other experiments (Hill & Bünger 2004). If many genes affect a trait, changes in gene frequency under selection are small, so variance is expected to change only slowly (Falconer & Mackay 1996); reductions in variance from those initially at high frequency may be largely compensated by increases in those initially rare; the influence of epistasis on response appears to be small (Crow 2008); and new models provide rationale (Barton & de Vladar 2009). Under the infinitesimal model, the total response deriving from the initial variation is expected to total 2*N*_e_ times the response in each early generation (Robertson 1960). The new variation arising from continuous mutation, an increment in heritability of the order of 0.1 per cent per generation, implies that substantial continued responses can also be achieved from mutations. This has been demonstrated in selection experiments from inbred bases (Hill 1982; Keightley & Halligan 2009), although many mutations revealed in selection experiments are retained only because their effects on the selected trait outweigh those on viability or fertility (López & López-Fanjul 1993). Taken together, Walsh (2004) showed that the response in the Illinois lines was mainly contributed by variation in the founder lines, but must have been due partly to mutations arising subsequently.

Modern breeding programmes inevitably involve a concentration of improvement in populations of limited size so that effective multi-trait recording can be undertaken and intense selection practised. There is a multiplication pyramid from nucleus populations in poultry and pigs, and in dairy cattle a concentration through use of sires through artificial insemination worldwide. Breeding programmes can be designed to optimize the trade-off between high selection intensity with the use of relatives' information to increase short-term gain and the decrease in *N*_e_ and likely long-term progress (Villanueva *et al*. 2006). But are there problems?

For cattle there is evidence that population sizes were large following domestication, of the order of tens of thousands or more, but those in some modern breeds are of the order of 100. Even so the levels of molecular genetic diversity within breeds are at least as great as in human populations (Gibbs *et al*. 2009). Nucleus populations of chickens likely have similar effective sizes. An analysis by Muir *et al*. (2008) of a large collection of lines of broilers, layers and those maintained by fanciers indicated that about one-half of the alleles present in Red Jungle Fowl, regarded as the progenitor native population, had been lost, with most of it occurring in early years of domestication. Yet, heritability remains high, indeed that for body weight seems to maintain its traditional value of about 25 per cent regardless. Further, each year over 40 × 10^9^ chickens are raised so, with a mutation rate of 1.8 × 10^−9^, there are over 50 mutants at each DNA site. The problem is not that there is no new variation, but to identify the useful new variants. Although it would be impossible to identify a mutant for a quantitative trait such as body weight in birds down the multiplication pyramid, it might be possible for a disease-resistant mutant.

We can be optimistic about the prospects for future improvement, not least because the input of molecular and high-throughput technologies to livestock improvement has so far been tiny. Clearly, there are limits imposed by the laws of thermodynamics, but by simply increasing the rate of live-weight gain of a bird, the efficiency of feed use is increased and also, a new consideration, greenhouse gas emissions per unit product is reduced. There are undoubtedly challenges, for example in the availability of water and climate change influences more generally, but new opportunities will come from new technology. Some, for example genomic selection, are really just extensions of classical quantitative genetic methods of increasing accuracy of selection. Others, for example changing or inserting new genes, provide radical ways of introducing new variation, but only if the public accepts them. Although conserved animal germ plasm far behind the commercial norm may harbour useful variants, I expect their contribution to genetic improvement to be small.

Similarly, the large amounts of genetic variation found in natural populations show that traits can be changed rapidly and substantially as a consequence of natural selection. With fitness defined as some simple measure, like bristle number in *Drosophila*, the effectiveness is illustrated by the results of many selection experiments (e.g. Weber 2004). There are also cases where fitness profiles and subsequently traits have changed greatly as a consequence of environmental change; for example, size of guppies increased substantially after transfer from a high to low predation environment, at rates similar to those found in laboratory selection experiments (Reznick *et al*. 1997). Although additive genetic variation and directional selection for particular traits have been shown, rarely have direct observations of natural populations revealed evolutionary changes, and those where responses were as expected were restricted to changes over one generation (Merila *et al*. 2001).

The ability to evolve depends on the additive genetic covariance structure of all the relevant traits, and whether the relevant combination actually expresses genetic variation. Recent analyses on genetic covariance matrices typically find that many of their eigenvalues are zero, such that the corresponding eigenvectors indicate directions of no variance (e.g. Blows & Walsh 2009; Kirkpatrick 2009; Walsh & Lynch 2009, ch. 30), which *if* these coincide with fitness ‘objectives’, implies adaptive evolution is not possible. Although these analyses indicate there are, indeed, trajectories that cannot be followed, sampling errors alone can lead to such inferences. To understand and predict changes or lack thereof, we greatly need more reliable information on the genetic covariances among multiple traits and on fitness profiles on many environments, but this is a massive task. In the presence of a major change of environment where fitness profiles change, the risk to a species seems more likely to come from other species-filling niches or evolving more rapidly rather than from its total inability to adapt.

## 9. Concluding remarks

Our level of understanding of many features of quantitative traits is quite rudimentary: what the genes do and how they interact, how their effects are distributed, the extent and magnitude of pleiotropic effects, the relations to overall fitness, and how and why is so much variation maintained? At this stage, however, we find that the many classical genes of small effect model explains many of the phenomena we observe and provides a basis for predictions of change. We can and are using the new information we get, however. But we should bear in mind that, as Darwin perceived, evolution succeeds through simple selection.
